# Inactivated Cells and Metabolites of *Saccharomyces boulardii* Alleviate Inflammation Damage in Caco-2 Monolayer Cells and Mice with Ulcerative Colitis

**DOI:** 10.3390/antiox14060737

**Published:** 2025-06-16

**Authors:** Yuxin Jin, Zehui Niu, Menglin Feng, Huilian Che, Zhihong Liang

**Affiliations:** 1 College of Food Science and Nutritional Engineering, China Agricultural University, Beijing 100083, China; j17860722880@163.com (Y.J.); hyeu2023@outlook.com (Z.N.); 18981579878@163.com (M.F.); 2 Sichuan Chengdu Advanced Agricultural & Industrial Institute, China Agricultural University, Chengdu 611430, China; 3 Beijing Laboratory for Food Quality and Safety, College of Food Science and Nutritional Engineering, China Agricultural University, Beijing 100083, China

**Keywords:** *Saccharomyces boulardii*, heat-inactivated cells, cell-free supernatant, anti-inflammation, ulcerative colitis, intestinal epithelial barrier

## Abstract

*Saccharomyces boulardii* (*S. boulardii*) has attracted widespread attention due to its antimicrobial and anti-inflammatory properties. In this study, we prepared postbiotics from the heat-inactivated cells (HIC) and cell-free supernatant (CFS) of *S. boulardii*, with the important component L-arginine (Arg) from the metabolic products included as one of the experimental groups. The results showed that in LPS-stimulated Caco-2 cells, HIC, CFS, and Arg protect intestinal epithelial barrier integrity by inhibiting the expression of TNF-α, IL-1β, and IL-6 while enhancing the expression of occludin and ZO-1 proteins. In dextran sulfate sodium (DSS)-induced colitis mice, HIC, CFS, and Arg alleviate symptoms such as weight loss and colonic damage while suppressing the upregulation of pro-inflammatory factors and the downregulation of tight junction proteins. Moreover, these postbiotics help restore the gut microbiota composition and functionality in colitis mice, with potentially superior regulatory effects compared to sulfasalazine (SASP). Overall, HIC and CFS protect the intestinal barrier function and improve DSS-induced colitis, supporting the development of functional food supplements.

## 1. Introduction

According to the latest estimates of the global burden of inflammatory bowel disease (IBD), the number of cases worldwide continues to rise, with the highest incidence observed in China and the United States [1]. This indicates that IBD remains a major public health concern globally. Ulcerative colitis (UC) is the primary type of IBD, characterized by persistent inflammation and ulceration of the colonic mucosa, leading to chronic diarrhea and rectal bleeding, which significantly impacts patients’ quality of life [2]. Currently, non-biological treatments such as aminosalicylates, corticosteroids, and immunomodulators are the main therapeutic options for UC [3]. However, relapse is common after medication treatment. Frequent drug therapy not only jeopardizes patients’ health but also imposes a significant economic burden. Since the mechanisms underlying UC and the factors contributing to relapse remain unclear, this has driven in-depth research into its pathological mechanisms and stimulated the demand for novel therapeutic approaches [4,5,6]. Consequently, efficient, economical, and safe functional foods are receiving increasing attention as an important direction for addressing this issue.

*Saccharomyces boulardii* (*S. boulardii*) is an important probiotic widely used in the food industry [7,8,9]. It enhances the flavor and taste of foods and improves products’ nutritional value and health benefits. Dairy products containing *S. boulardii* have demonstrated significant probiotic activity, which is beneficial for gut health [10]. Additionally, *S. boulardii* exhibits anti-cancer, anti-bacterial, and anti-viral activities, making it useful in treating gastrointestinal diseases [11,12]. However, probiotics are strain-specific [13], and their effects on the gut depend on their colonization rates and survival [14]. Postbiotics can address these limitations of probiotics and are considered a safer novel alternative [15]. Inactivated probiotic cells, metabolic products, or components of microbial cells can be referred to as postbiotics, provided they benefit health [16]. Many studies have explored the anti-inflammatory effects of postbiotics derived from *S. boulardii*, but detailed investigations into its components are still lacking [17,18]. To address this gap, this study will separately prepare the cell components and metabolic products of *S. boulardii* and comprehensively investigate the effects of inactivated *S. boulardii* cells and their metabolic products (collectively referred to as *S. boulardii* postbiotics) on regulating intestinal inflammation both in vitro and in vivo. Additionally, although there have been reports on the antioxidant properties of *S. boulardii* [19,20,21], research on the antioxidant capacity of postbiotics remains limited. Therefore, before investigating their anti-inflammatory effects, we also evaluated the antioxidant capacity of the two prepared postbiotics to comprehensively explore their potential.

UC primarily affects the colon. Its pathogenesis is characterized by a marked downregulation of tight junction proteins (TJPs), such as ZO-1 and occludin, in intestinal epithelial cells, thereby compromising the integrity of the intestinal epithelial barrier. Regarding this characteristic, we employed Caco-2 cells to model the intestinal epithelial barrier in vitro and established a DSS-induced UC mouse model. By evaluating the effects of inactivated *S. boulardii* cells and their metabolic products in protecting the intestinal epithelial barrier, reducing inflammation, and modulating the gut microbiota, this study will provide a theoretical basis for the potential application of postbiotics in functional foods while also offering more treatment options for patients with ulcerative colitis.

## 2. Materials and Methods

### 2.1. Main Reagents and Materials

Fetal bovine serum (FBS), Dulbecco’s modified eagle medium (DMEM), penicillin-streptomycin (P/S), phosphate-buffered saline (PBS), lipopolysaccharide (LPS), cell counting kit-8 (CCK-8), alkaline phosphatase assay kit, and QuickBlock™ blocking buffer were purchased from Thermo Fisher Scientific Inc. (Waltham, MA, USA). Antibodies, including ZO-1, occludin, GAPDH, and goat anti-rabbit IgG(H + L) secondary antibody, as well as ELISA kits for human TNF-α, IL-1β, IL-6, and mouse TNF-α, IL-1β, IL-6, alkaline phosphatase assay kit were purchased from Beyotime Biotechnology Co., Ltd. (Shanghai, China). L-arginine (Arg) was purchased from Shanghai Aladdin Biochemical Technology Co., Ltd. (Shanghai, China). RIPA buffer, protease inhibitor, PMSF, and 4% paraformaldehyde were obtained from Solarbio Science & Technology Co., Ltd. (Beijing, China). SuperKine™ west pico plus chemiluminescent substrate (ECL) was purchased from Abbkine Scientific Co., Ltd. (Wuhan, China). SuperReal PreMix Plus (SYBR Green) was from Tiangen Biochemical Technology Co., Ltd. (Beijing, China). TransZolTM up plus RNA kit and TransScript^®^ one-step gDNA removal and cDNA synthesis superMix were purchased from TransGen Biotech Co., Ltd. (Beijing, China). DSS was obtained from Yisheng Biotechnology Co., Ltd. (Shanghai, China).

### 2.2. S. Boulardii Metabolite Analysis

The *S. boulardii* strain was obtained from the laboratory collection. *S. boulardii* was inoculated at 1% (*v*/*v*) into 100 mL of YPD liquid medium and cultured at 28 °C with 200 rpm for 12 h, reaching the stationary phase (approximately 10^8^ CFU/mL). The culture was transferred to a 50 mL centrifuge tube and then centrifuged at 6000 rpm, 4 °C, for 10 min, and the supernatant was filtered through a 0.22 μm membrane. A volume of 100 µL of cell-free supernatant (CFS) and YPD liquid sample was placed into separate 1.5 mL centrifuge tubes, and then a volume of 400 μL of extraction solution (acetonitrile to methanol = 1:1) was added. The sample was vortexed for 30 s and then transferred to an ice-water bath and sonicated for 10 min. After sonication, the sample was incubated at −20 °C for 30 min. Following incubation, the sample was centrifuged at 12,000× *g* for 15 min at 4 °C. The supernatant was carefully collected and transferred to a nitrogen evaporator, where it was evaporated to near dryness under a gentle nitrogen stream. An amount of 100 µL of reconstitution solution (acetonitrile to water = 1:1, *v*/*v*) was added to the dried residue and sonicated for 5 min to ensure complete dissolution. Then, the centrifugation step (12,000× *g*, 4 °C, 15 min) was repeated, and the supernatant was transferred from the second centrifugation to a clean injection vial for liquid chromatography-mass spectrometry (LC-MS) analysis. To ensure experimental stability, equal volumes of all sample supernatants were mixed to prepare a quality control (QC) sample for method precision validation. The mixing process was performed using a pipette for precise aliquoting, followed by vortexing for 10 s to ensure homogeneity.

### 2.3. Preparation of Postbiotics

The *S. boulardii* cells were heat-inactivated at 100 °C for 30 min, washed with PBS, and ground using a cryogenic grinder (Retsch Cryomill, Verder Instrument & Equipment Co., Ltd., Shanghai, China) (20 m/s, 5 min). The ground heat-inactivated cells (HIC) and the CFS were freeze-dried for 48 h and prepared into powders for subsequent studies.

### 2.4. Caco-2 Cell Culture and Viability Assay

The human colon cancer cell line Caco-2 was obtained from the Type Culture Collection of the Chinese Academy of Sciences (Shanghai, China). Caco-2 cells were cultured in a complete medium (DMEM supplemented with 20% FBS and 1% P/S) under 37 °C 5% CO_2_. Caco-2 cells were seeded into a 96-well plate (5000 cells/100 μL) and cultured for 24 h until most cells adhered to the surface. Then, the medium was replaced with a serum-free medium and cultured for 12 h. After removing the medium, 100 μL of different concentrations (0, 0.5, 5, 50, 500, 5000 μg/mL) of HIC, CFS, and Arg were added to each well, and cells were co-cultured for 24 and 48 h. After 24 and 48 h, cell viability was measured using the CCK-8 assay, and absorbance (OD) values were recorded at 450 nm using a microplate reader. The cell viability (%) is defined as the ratio of the number of viable cells in the experimental group to that in the control group, where the cell viability in the control group is considered 100%.

### 2.5. Measurement of Antioxidant Capacity

The method is from reference [22]. A volume of 200 µL of 0.1 mmol/L DPPH absolute ethanol solution was added into the detection wells of a 96-well plate. Then, a volume of 10 µL of probiotics at different concentrations (0, 0.5, 5, 50, 500, 5000 μg/mL) was added, respectively. After gently shaking and mixing it evenly, it was incubated at 37 °C for 30 min. After the reaction was completed, the absorbance was measured at a wavelength of 517 nm. DPPH radical retention rate (%) = A1/A0 × 100, where A1 is the absorbance of postbiotics at concentrations ranging from 0.5 to 5000 μg/mL, and A0 is the absorbance at a postbiotic concentration of 0.

Equal volumes of a 7.4 mmol/L ABTS solution and a 2.6 mmol/L potassium persulfate solution were mixed. After keeping it in the dark for 12 h, it was diluted with absolute ethanol until the absorbance at 734 nm was 0.7 ± 0.05 to obtain the ABTS working solution. A volume of 200 µL of the ABTS working solution was added into the detection wells of a 96-well plate. Then, a volume of 10 µL of postbiotics at different concentrations (0, 0.5, 5, 50, 500, 5000 μg/mL) was added, respectively. After gently shaking and mixing it evenly, the mixture was incubated at 37 °C for 6 min. After the reaction was completed, the absorbance was measured at a wavelength of 734 nm. ABTS radical retention rate (%) = A1/A0 × 100, where A1 is the absorbance of postbiotics at concentrations ranging from 0.5 to 5000 μg/mL, and A0 is the absorbance at a postbiotic concentration of 0.

### 2.6. Measurement of Transepithelial Electrical Resistance (TEER)

The method of constructing the Caco-2 cell monolayer model refers to previous studies [23,24]. Caco-2 cell suspension (2 × 10^4^ cells/200 μL) was seeded into the apical side (AP) of polyester membrane inserts with a pore size of 0.4 μm in a 24-well Transwell plate (Corning Inc., Kennebunk, ME, USA), with 500 μL of complete medium (DMEM supplemented with 20% FBS and 1% P/S) added to the basolateral side (BL). After seeding, the culture medium was replaced every other day for the first 7 days and then daily for 21 days of continuous culture. Electrical resistance was measured every three days. The TEER was calculated using the formula: TEER = (measured resistance of the well-blank well resistance) Ω × 0.33 cm^2^. When the TEER value stabilized and >400 Ω·cm^2^, the medium was replaced with a serum-free medium and cultured for 12 h. Subsequently, the control group (complete medium), the LPS group (100 μg/mL LPS), and the HIC, CFS, and Arg groups (100 μg/mL LPS + 500 μg/mL HIC, CFS, and Arg, respectively) were treated for 24 h. Electrical resistance was measured using a Millicell-ERS2 volt-ohmmeter (Millipore, Bedford, MA, USA).

### 2.7. Measurement of Alkaline Phosphatase (ALP) Activity

This study evaluated the integrity and functionality of intestinal epithelial cells by measuring ALP activity in the culture media on both sides of the Caco-2 cell membrane [25,26]. The establishment and treatment of the Caco-2 cell monolayer model followed the method described in Section 2.6. When the TEER value stabilized and >400 Ω·cm^2^, the complete medium was replaced with serum-free medium without FBS and cultured for 12 h. Subsequently, the culture medium on the AP side was replaced with the following treatments: the control group (complete medium only), the LPS group (complete medium containing 100 µg/mL LPS), and the HIC, CFS, and Arg groups (complete medium containing 100 µg/mL LPS + 500 μg/mL HIC, CFS, and Arg, respectively). The culture medium on the BL side was uniformly replaced with serum-free medium without FBS for all groups. After 24 h of treatment, 200 µL of culture supernatant were collected from both the AP and BL sides of the 24-well Transwell plate for ALP activity measurement. ALP activity was measured according to the instructions of the ALP assay kit by recording absorbance at 405 nm, and the ALP activity in both the AP and BL side culture media was determined. The ALP ratio (AP/BL) was then calculated.

### 2.8. Animals and Animal Experiment Design

Specific pathogen-free (SPF) male C57BL/6J mice (6–8 weeks old, 20–24 g) were obtained from Beijing Vital River Laboratory Animal Technology Co., Ltd. (SCXK (Beijing) 2021-0006). Mice were housed in a standard SPF facility under controlled conditions (22 ± 2 °C, 40–70% humidity, 12 h light/dark cycle) with four mice per ventilated cage.

After a week of acclimatization to the experimental environment, 48 male C57BL/6J mice were divided into six groups (*n* = 8): the normal control group (CON), colitis model group (DSS), HIC treatment group (HIC), CFS treatment group (CFS), sulfasalazine (SASP) treatment group (SASP), and Arg treatment group (Arg). All other groups were given free access to 2.5% DSS for one week except for the CON group. The DSS solution was refreshed daily. From the eighth day, DSS was discontinued for all groups, and from days 8 to 14, all groups were given distilled water. Meanwhile, mice in the HIC, CFS, Arg, and SASP groups were gavaged with 200 µL of 200 mg/kg heat-inactivated cells of *S. boulardii*, 200 mg/kg cell-free supernatant of *S. boulardii*, 200 mg/kg Arg, and 200 mg/kg SASP, respectively, for one week. This dosage is based on the typical administration concentration of SASP [27,28]. All substances were dissolved in sterile PBS. Mice in the CON and DSS groups were gavaged daily with 200 µL of sterile PBS. From day 8, the treatment groups received gavage administration every other day (on days 9, 11, and 13), with sterile PBS administered on other days. Mice disease activity index (DAI) was evaluated based on the criteria in the Supplementary Table S1. On day 15, blood samples were collected by eye blood sampling, allowed to stand at room temperature for 2 h, and then centrifuged at 3500 rpm for 15 min at 4 °C to obtain serum. Serum samples were stored at −80 °C. Following blood collection, the mice were euthanized by cervical dislocation and immediately dissected. The spleen, liver, and kidneys were weighed to calculate the organ index (organ weight/body weight × 100). The entire colon was excised, and its length measured. The distal colon was fixed in 4% paraformaldehyde, while the remaining colon and cecal contents were stored at −80 °C for further analysis.

### 2.9. Histopathological Analysis

A 1–2 cm segment of the colon was taken, then dehydrated with ethanol, cleared with xylene, and then embedded in paraffin. The tissue was sectioned, deparaffinized, and rehydrated. Afterward, it was stained with hematoxylin and eosin (H&E) and finally mounted for microscopic observation. Based on reference [29], the scoring criteria for colon tissue pathology induced by DSS start from 0, with higher scores indicating more severe colon damage, and the maximum score is 11.

### 2.10. Measurement of Pro-Inflammatory Cytokine

A volume of 200 µL of Caco-2 cell suspension (approximately 2 × 10^4^ cells) was inoculated into a 24-well Transwell plate and continuously cultured for 21 days. When the TEER value of Caco-2 cells reached 400 Ω·cm^2^, the cells were cultured in a serum-free medium for 12 h. The CON group (complete medium), the LPS group (100 μg/mL LPS), and the HIC, CFS, and Arg groups (100 μg/mL LPS + 500 μg/mL HIC, CFS, or Arg, respectively) were treated for 24 h. Subsequently, the culture supernatant from the AP side of the Transwell plate was collected. The levels of pro-inflammatory cytokines (TNF-α, IL-1β, and IL-6) in the cell culture supernatant and mouse serum were detected using human and mouse ELISA kits, respectively, following the manufacturer’s instructions.

### 2.11. Measurement of mRNA Expression

RNA was extracted from Caco-2 cells (monolayer cells differentiated after 21 days) and colon tissues using the TransZolTM up plus RNA kit, followed by reverse transcription into cDNA. Quantitative real-time PCR (RT-qPCR) was then performed using SuperReal PreMix Plus (SYBR Green) to assess the mRNA expression levels of *TNF-α*, *IL-1β*, *IL-6*, *occludin*, and *ZO-1* in both Caco-2 cells and colon tissues, normalized to *GAPDH* expression. Primer sequences for human and mouse genes are listed in Supplementary Tables S2 and S3.

### 2.12. Measurement of Protein Expression

Protein expression of occludin and ZO-1 in Caco-2 cells (monolayer cells differentiated after 21 days) and colon tissues was measured using Western blotting. Proteins were extracted with RIPA buffer, protease inhibitor, and PMSF (100:1:1), followed by centrifugation at 12,000× *g* for 10 min at 4 °C. SDS-PAGE was used to separate the proteins, which were then transferred to a PVDF membrane and blocked with 5% skim milk for 1 h. The membrane was incubated overnight at 4 °C with primary antibodies: occludin (1:1000) and ZO-1 (1:1000). After washing with TBST, a goat anti-rabbit IgG HRP-conjugated secondary antibody (1:2000) was applied for 1 h. Following further washes, proteins were detected using ECL and visualized with a chemiluminescence imaging system. GAPDH was used as an internal control. The relative protein expression was quantified using ImageJ (V1.8.0) by calculating the ratio of the target protein intensity to GAPDH.

### 2.13. Analysis of Gut Microbiota

DNA was extracted from fecal samples using QIAamp DNA isolation kits (Hilden, Germany). The V3–V4 region of the 16S rRNA gene was amplified with primers 338F (5′-ACTCCTACGGGAGGCAGCAG-3′) and 806R (5′-GGACTACHVGGGTWTCTAAT-3′), and sequencing was performed on the Nova-Seq platform (Illumina, San Diego, CA, USA). Raw data were filtered and analyzed with QIIME (v1.9.1), and OTUs were clustered at >97% similarity using UPARSE (v7.0.1001). α and β diversity was calculated in QIIME, and PCoA and NMDS plots were generated on the Majorbio Cloud Platform using the weighted-unifrac distance algorithm. Linear discriminant analysis (LDA) with LDA effect size (LEfSe) was used to identify differentially abundant taxa (*p* < 0.05, LDA score > 4). PICRUSt predicted microbial functional features, while STAMP (v2.1.3) was used to visualize statistical differences in these features across samples.

### 2.14. Statistical Analysis

The data, presented as means ± SD, were analyzed using GraphPad Prism 9.0 software (GraphPad Software, San Diego, CA, USA). Prior to selecting the appropriate ANOVA method, normality testing was rigorously conducted to ensure the validity of the statistical approach. Statistical significance was evaluated by one-way or two-way ANOVA, with Duncan’s test. *p* < 0.05 was considered statistically significant, and *p* < 0.01 was considered to be critically significant.

## 3. Results

### 3.1. Main Components of S. Boulardii Metabolites

**Figure 1:**
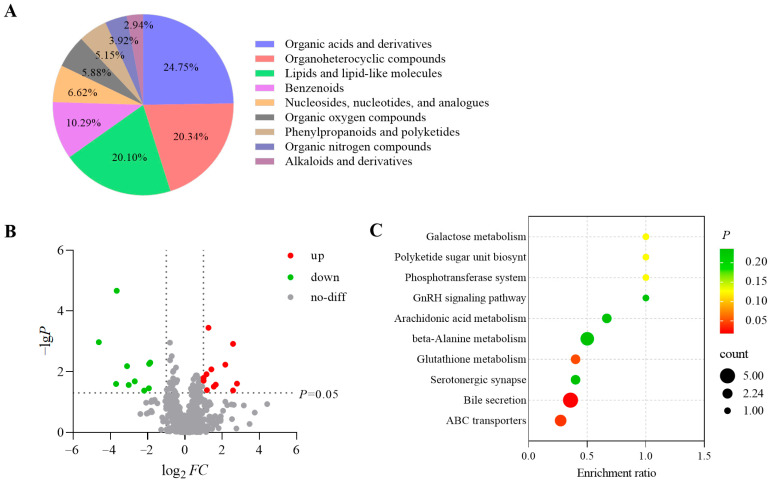
*S. boulardii* metabolite analysis. (**A**) Proportional distribution of various metabolites. (**B**) Volcano plot of differential metabolites between CFS and YPD. (**C**) KEGG enrichment bubble plot of differential metabolites in CFS.

We used LC-MS to identify nine major classes of *S. boulardii* metabolites, with organic acids and derivatives accounting for the largest proportion (24.75%) (Figure 1A). Among these, amino acids drew our attention. Among the identified amino acids, seven L-amino acids were found to be directly involved in human protein synthesis. Based on the relative quantification data, Arg exhibited the highest relative abundance (Table 1). Additionally, differential analysis between CFS and YPD identified 12 upregulated metabolites in CFS, including adenosine, leucyl-proline, and arginine (Figure 1B, Table S4). Based on these findings, we selected Arg as a research target to assess its function and potential applications in CFS. Further pathway enrichment analysis revealed that the most significantly enriched pathways in CFS compared to YPD were bile secretion, ABC transporters, and glutathione metabolism (Figure 1C), suggesting that *S. boulardii* metabolites are more complex than the culture medium and may have potential biological benefits.

### 3.2. S. Boulardii Postbiotics Have Antioxidant Capacity

**Figure 2:**
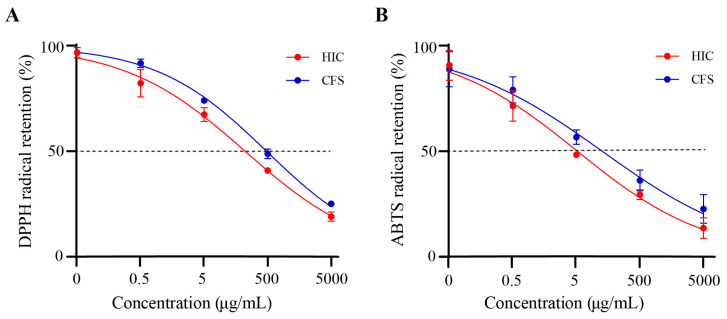
Antioxidant capacity tests of HIC and CFS. (**A**) DPPH radical retention of HIC and CFS (0.091 mM DPPH). (**B**) ABTS radical retention of HIC and CFS.

As the concentrations of HIC and CFS increase, the retention rates of both DPPH radicals and ABTS radicals decrease (Figure 2). That is to say, the radical scavenging capabilities of HIC and CFS increase with the rise in concentration. Specifically, the IC50 values of the DPPH radical scavenging rates for HIC and CFS are 220.6 μg/mL and 479.9 μg/mL, respectively, and the IC50 values of the ABTS radical scavenging rates for HIC and CFS are 51.89 μg/mL and 127.4 μg/mL, respectively. This indicates that HIC has a stronger antioxidant capacity than CFS.

### 3.3. S. Boulardii Postbiotics Improve the TEER and ALP Activity of the Caco-2 Cell Monolayer

**Figure 3:**
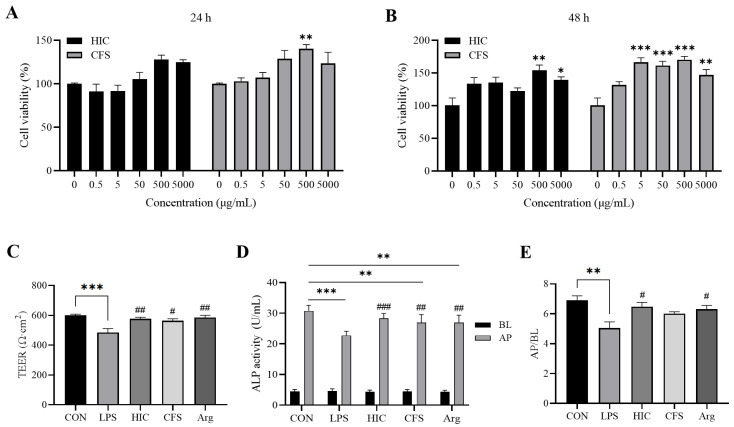
Effects of HIC, CFS, and Arg on Caco-2 cells’ TEER and ALP activity. Cell viability after (**A**) 24 h and (**B**) 48 h of co-culture. (**C**) TEER. (**D**) ALP activity. (**E**) AP/BL. Note: Comparison of significant differences between groups is indicated, where * *p* < 0.05, ** *p* < 0.01, *** *p* < 0.001 vs. CON; # *p* < 0.05, ## *p* < 0.01, and ### *p* < 0.001 vs. LPS.

The postbiotics of *S. boulardii* at different concentrations can promote cell viability, with the optimal concentration being 500 μg/mL (Figure 3A,B). Combined with the antioxidant activity results of both HIC and CFS, we selected a uniform concentration of 500 μg/mL for both postbiotics to investigate their anti-inflammatory effects on Caco-2 cells. Additionally, we included the key active component, Arg, from CFS as an intervention group to evaluate its potential role in anti-inflammatory activity. After LPS treatment, the TEER of the Caco-2 cell monolayer in the LPS group was significantly reduced (*p* < 0.001), while HIC, CFS, and Arg significantly alleviated this reduction (*p* < 0.05) (Figure 3A). ALP exhibited a highly asymmetric distribution between the AP and BL sides of the cell monolayer, demonstrating clear polarization (Figure 3B). Compared to the CON group, the AP/BL in the LPS group significantly decreased after LPS induction (*p* < 0.01). Both HIC and Arg significantly improved this decrease (*p* < 0.05) (Figure 3C). CFS treatment also increased the AP/BL, but the rise was not statistically significant compared to the LPS group (*p* > 0.05).

### 3.4. S. Boulardii Postbiotics Inhibit the Secretion of Pro-Inflammatory Cytokines by Caco-2 Cell Monolayers

**Figure 4:**
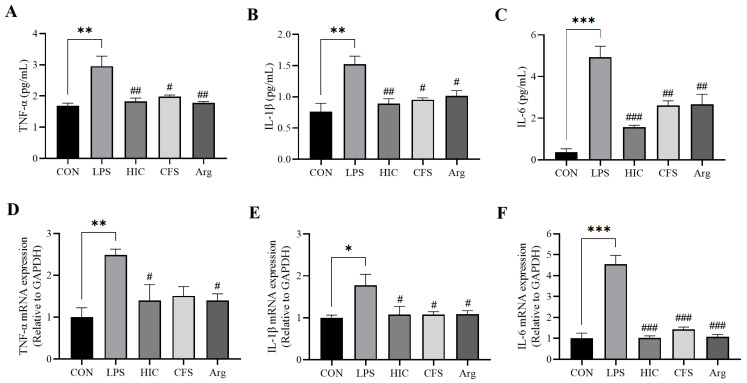
Effects of HIC, CFS, and Arg on the secretion of pro-inflammatory cytokines by Caco-2 cells. The levels of (**A**) TNF-α, (**B**) IL-1β, and (**C**) IL-6 in the Caco-2 cell culture supernatant. The mRNA expression levels of (**D**) *TNF-α*, (**E**) *IL-1β*, and (**F**) *IL-6* in Caco-2 cells. Note: Comparison of significant differences between groups is indicated, where * *p* < 0.05, ** *p* < 0.01, *** *p* < 0.001 vs. CON; # *p* < 0.05, ## *p* < 0.01, and ### *p* < 0.001 vs. LPS.

After LPS treatment, the levels of TNF-α, IL-1β, and IL-6 in the Caco-2 cell monolayer of the LPS group were significantly elevated (*p* < 0.01). HIC, CFS, and Arg were able to significantly reduce the secretion of these pro-inflammatory cytokines (*p* < 0.05) (Figure 4A–C). At the mRNA expression level, the expressions of *TNF-α*, *IL-1β*, and *IL-6* were also significantly increased in the LPS group (*p* < 0.05), while both HIC and Arg significantly downregulated the mRNA expression of these three cytokines (*p* < 0.05). CFS also significantly reduced the expression of *IL-1β* and *IL-6* (*p* < 0.05), but its inhibition of *TNF-α* did not show a statistically significant difference compared to the LPS group (*p* > 0.05) (Figure 4D–F).

### 3.5. S. Boulardii Postbiotics Protect TJPs of Caco-2 Cell Monolayers

**Figure 5:**
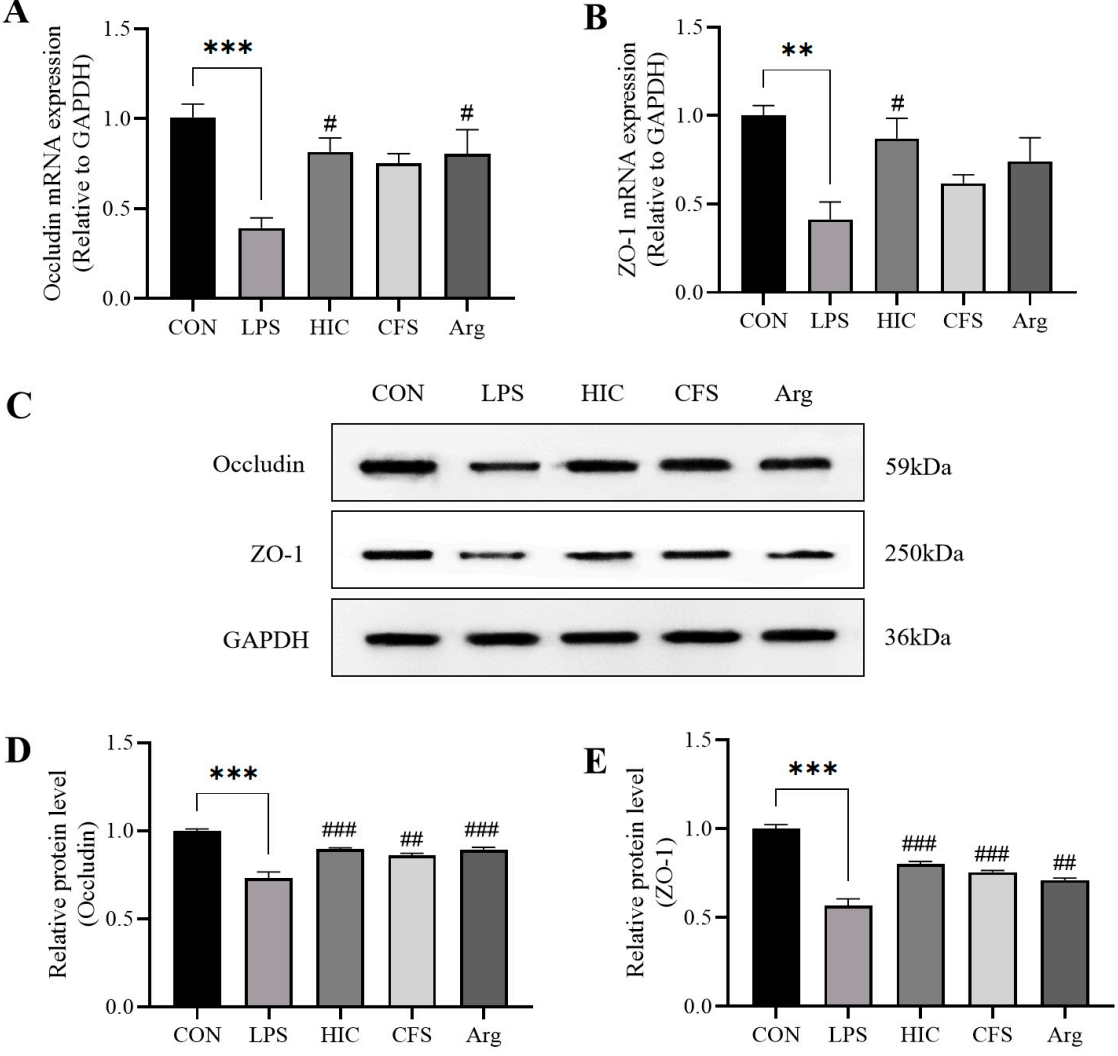
Effect of HIC, CFS, and Arg on the levels of occludin and ZO-1 in the Caco-2 cell monolayer model. Relative mRNA expression of (**A**) *occludin* and (**B**) *ZO-1*. (**C**) Western blotting images of occludin and ZO-1 protein expression. Relative protein expression of (**D**) occludin and (**E**) ZO-1. Note: Comparison of significant differences between groups is indicated, where ** *p* < 0.01, *** *p* < 0.001 vs. CON; # *p* < 0.05, ## *p* < 0.01, and ### *p* < 0.001 vs. LPS.

We evaluated occludin and ZO-1 at transcriptional and translational levels. At the mRNA level, the expression of *occludin* and *ZO-1* was significantly reduced in the LPS group (*p* < 0.01). HIC treatment significantly increased the mRNA expression of TJPs (*p* < 0.05). Arg only significantly improved *occludin* mRNA expression (*p* < 0.05), while CFS had no significant effect on the recovery of either protein (*p* > 0.05) (Figure 5A,B). Western blot analysis further confirmed that LPS treatment markedly reduced occludin and ZO-1 protein expression in the LPS group (*p* < 0.001). However, postbiotics (HIC, CFS) and Arg were able to significantly restore the expression of both proteins (*p* < 0.01) (Figure 5C–E).

### 3.6. S. Boulardii Postbiotics Alleviate the Conventional Pathological Indicators in Colitis Mice

**Figure 6:**
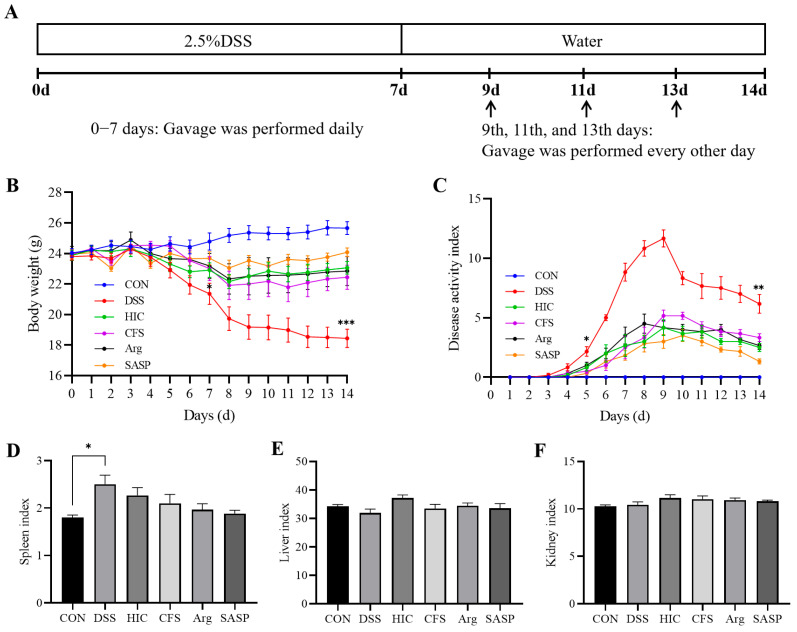
Effect of HIC, CFS, Arg, and SASP on conventional pathological indicators in colitis mice. (**A**) The animal experimental design. (**B**) Body weight curve. (**C**) DAI curve. (**D**) Spleen index. (**E**) Liver index. (**F**) Kidney index. Note: Comparison of significant differences between groups is indicated, where * *p* < 0.05, ** *p* < 0.01, *** *p* < 0.001 vs. CON.

We established a mouse model of ulcerative colitis using 2.5% DSS, with SASP as a positive control. We aimed to study the in vivo anti-inflammatory and protective effects on the intestinal epithelial barrier of HIC, CFS, and Arg. The experimental design is shown in Figure 6A. There was no significant difference in body weight among the groups during the first 6 days (*p* > 0.05). However, starting from day 7, the body weight of mice in the DSS group significantly decreased (*p* < 0.05), and by the end of the experiment, there was a highly significant difference in final body weight compared to the CON group (*p* < 0.001). The other four treatment groups were able to restore the body weight of DSS-induced colitis mice (Figure 6B). Regarding the DAI, the DSS group showed a significant difference from the CON group starting on day 5 (*p* < 0.05). Because the administration of DSS ceased after day 7, the DAI index of the DSS group began to decrease on day 9. Still, a highly significant difference from the CON group remained by the end of the study (*p* < 0.01). Consistent with body weight changes, the other four treatment groups alleviated the increase in the DAI index (Figure 6C). To evaluate the potential impact of HIC, CFS, and Arg on the function of major organs, the liver, spleen, and kidney indices were measured. The spleen index of the DSS group significantly increased (Figure 6D). There were no significant differences in liver and kidney indices among the groups (Figure 6E,F).

### 3.7. S. Boulardii Postbiotics Improve the Colon Condition in Colitis Mice

**Figure 7:**
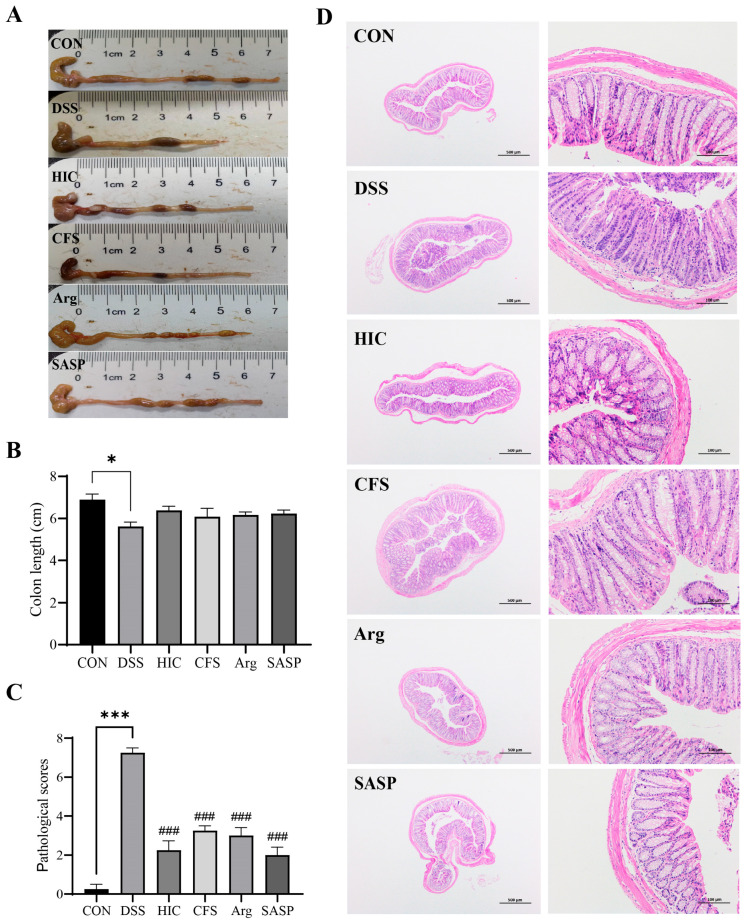
Effect of HIC, CFS, Arg, and SASP on the colon appearance and internal pathology in colitis mice. (**A**) Colon tissue. (**B**) Colon length. (**C**) Pathological scores. (**D**) H&E staining result (at 40 and 200 magnification). In the image, H&E stains the cell nuclei and cytoplasm (or extracellular matrix) in blue-purple and pink, respectively. Note: Comparison of significant differences between groups is indicated, where * *p* < 0.05, *** *p* < 0.001 vs. CON; ### *p* < 0.001 vs. DSS.

The colonic length in the DSS group was significantly shortened (*p* < 0.05), while HIC, CFS, Arg, and SASP effectively prevented the DSS-induced shortening of the colon (Figure 7A,B). H&E staining revealed that the colonic crypt structure was clear in healthy mice, and goblet cells were arranged in a tight, orderly fashion. The DSS group showed destroyed crypt architecture, a significant loss of goblet cells, and infiltration of inflammatory cells. In the treatment groups, the crypt structure was restored. Goblet cells were neatly arranged (Figure 7D). This indicates that treatment with HIC, CFS, and Arg can significantly alleviate the pathological damage and inflammation observed in the DSS group, with therapeutic effects comparable to the positive control SASP (*p* < 0.001) (Figure 7C).

### 3.8. S. Boulardii Postbiotics Inhibit Pro-Inflammatory Cytokines in Colitis Mice

**Figure 8:**
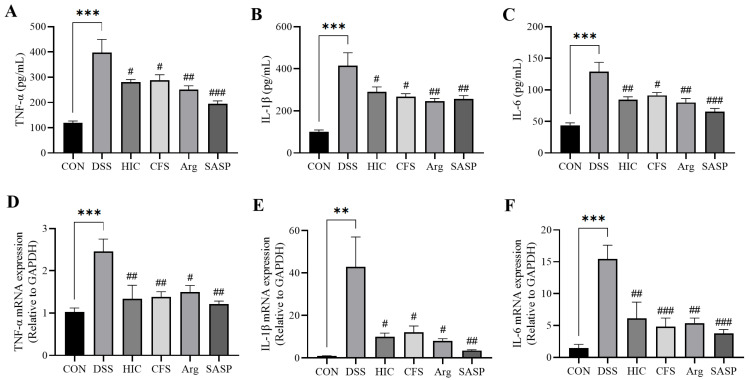
Effects of HIC, CFS, Arg, and SASP on pro-inflammatory cytokines in colitis mice. The levels of (**A**) TNF-α, (**B**) IL-1β, and (**C**) IL-6 in serum. The mRNA expression levels of (**D**) TNF-α, (**E**) IL-1β, and (**F**) IL-6 in colonic tissue. Note: Comparison of significant differences between groups is indicated, where ** *p* < 0.01, *** *p* < 0.001 vs. CON; # *p* < 0.05, ## *p* < 0.01, and ### *p* < 0.001 vs. DSS.

DSS treatment significantly increased the levels of TNF-α, IL-1β, and IL-6 in the blood of the DSS group mice (*p* < 0.001). Compared to the DSS group, the levels of TNF-α, IL-1β, and IL-6 in the HIC, CFS, Arg, and SASP groups were significantly reduced (*p* < 0.05) (Figure 8A–C). In terms of mRNA expression in the colon, the DSS group exhibited a marked increase in TNF-α, IL-1β, and IL-6 expression (*p* < 0.01). Consistent with the blood measurements, treatment with HIC, CFS, Arg, and SASP significantly decreased the mRNA expression of these three pro-inflammatory cytokines in the colon (*p* < 0.05) (Figure 8D–F). These findings suggest that in DSS-induced colitis mice, HIC, CFS, and Arg effectively reduce the expression of TNF-α, IL-1β, and IL-6 in both the blood and colonic tissues, with therapeutic effects comparable to those of the positive control, SASP.

### 3.9. S. Boulardii Postbiotics Protect TJPs in Colitis Mice

**Figure 9:**
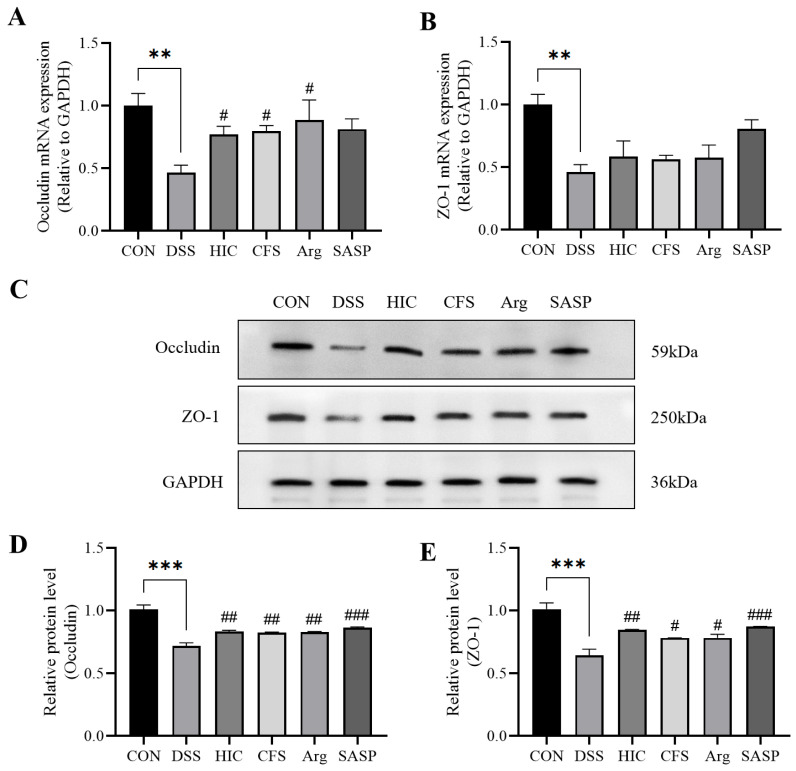
Effect of HIC, CFS, Arg, and SASP on the levels of occludin and ZO-1 in colitis mice. Relative mRNA expression of (**A**) occludin and (**B**) *ZO-1*. (**C**) Western blotting images of occludin and ZO-1 protein expression. Relative protein expression of (**D**) occludin and (**E**) ZO-1. Note: Comparison of significant differences between groups is indicated, where ** *p* < 0.01, *** *p* < 0.001 vs. CON; # *p* < 0.05, ## *p* < 0.01, and ### *p* < 0.001 vs. DSS.

At the mRNA expression level, occludin and *ZO-1* were significantly downregulated in the DSS treatment group (*p* < 0.01). Treatment with HIC, CFS, and Arg significantly increased occludin mRNA expression (*p* < 0.05) but had no notable effect on the recovery of ZO-1 expression (*p* > 0.05). Interestingly, SASP showed no significant restorative effect on either of these TJPs (*p* > 0.05) (Figure 9A,B). Further Western blot analysis revealed that DSS treatment markedly reduced the protein expression of occludin and ZO-1 in the DSS group (*p* < 0.001), while all treatment groups significantly restored their expression (*p* < 0.05) (Figure 9C–E). These findings indicate that HIC, CFS, and Arg can effectively recover the levels of TJPs in the colon of DSS-induced colitis mice.

### 3.10. Effect of S. Boulardii Postbiotics on the Overall Structure of the Gut Microbiota in Colitis Mice

**Figure 10:**
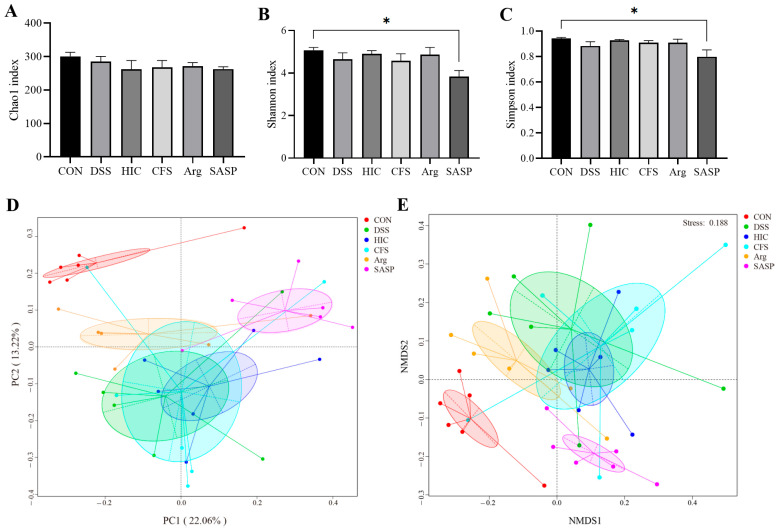
Effect of HIC, CFS, Arg, and SASP on the diversity and overall structure of the gut microbiota. (**A**) Chao1 index. (**B**) Shannon index. (**C**) Simpson index. (**D**) PCoA analysis. (**E**) NMDS analysis. Note: Comparison of significant differences between groups is indicated, where * *p* < 0.05 vs. CON.

α-diversity indices (Chao1, Shannon, and Simpson) and β-diversity analyses (PCoA and NMDS) were used to evaluate microbial alterations. DSS induction did not significantly affect the α-diversity of the gut microbiota in mice. However, compared to the CON group, SASP administration significantly reduced the Shannon and Simpson indices (*p* < 0.05), while no such differences were observed in the HIC, CFS, and Arg groups (Figure 10A–C). This suggests that SASP may exert a specific effect on the gut microbiota. The clustering results of PCoA and NMDS further support this hypothesis. The DSS group deviated from the CON group, while the HIC, CFS, and Arg groups clustered near the DSS group, with the Arg group being closest to the control. The SASP group formed a distinct cluster, indicating that SASP uniquely modulates the gut microbiota structure (Figure 10D,E). These results suggest that DSS induction and treatment with HIC, CFS, or Arg do not significantly alter the overall structure of the gut microbiota in colitis mice, while SASP treatment has a marked impact.

### 3.11. Effect of S. Boulardii Postbiotics on the Gut Microbiota at the Phylum and Genus Levels in Colitis Mice

**Figure 11:**
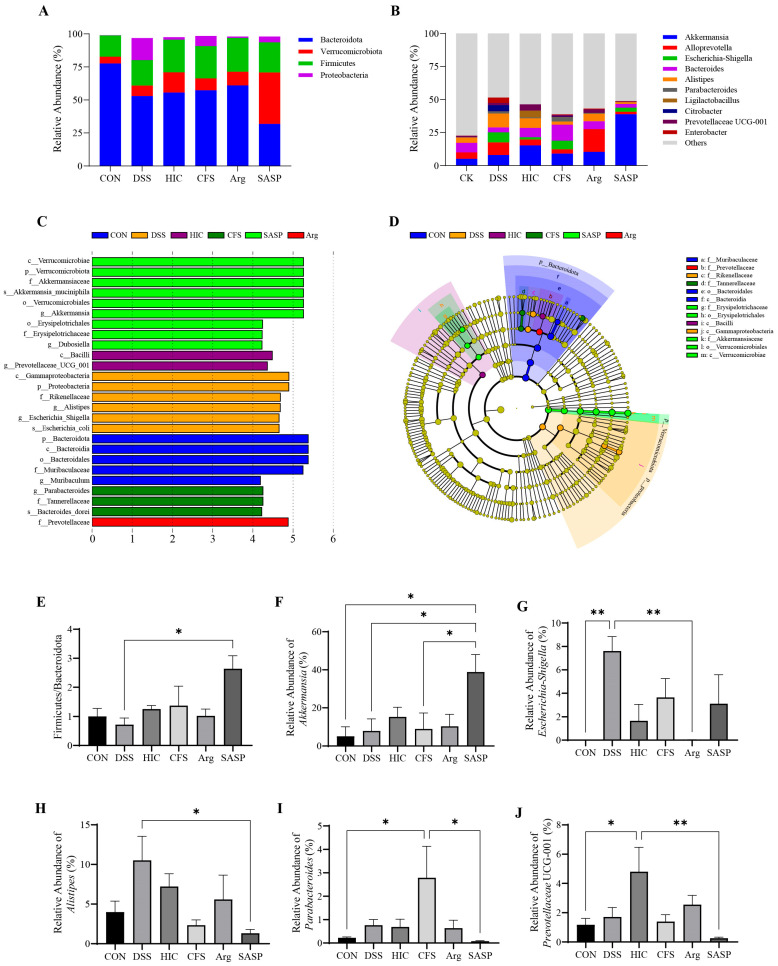
Effect of HIC, CFS, Arg, and SASP on the gut microbiota composition in colitis mice. (**A**) The relative abundance at the phylum level. (**B**) The relative abundance at the species level. (**C**) Indicator bacteria with LDA scores of >4. (**D**) LEfSe Cladogram. (**E**) Firmicutes/Bacteroidota. (**F**) Comparison of relative abundance differences of *Akkermansia* between each group and the SASP group. (**G**) Comparison of relative abundance differences of *Escherichia-Shigella* between each group and the DSS group. (**H**) Comparison of relative abundance differences of *Alistipes* between each group and the DSS group. (**I**) Comparison of relative abundance differences of Parabacteroides between each group and the CFS group. (**J**) Comparison of relative abundance differences of *Prevotellaceae* UCG-001 between each group and the HIC group. Note: Comparison of significant differences between groups is indicated, where * *p* < 0.05, ** *p* < 0.01.

At the phylum level, the gut microbiota of the mice mainly consist of four phyla, Bacteroidota, Verrucomicrobiota, Firmicutes, and Proteobacteria, which account for over 99% of the gut microbiota. Among these, the Bacteroidota phylum comprises over 50%, indicating its significant regulatory role in the mouse gut (Figure 11A). Compared to the CON group, the SASP group significantly reduced the abundance of this phylum (*p* < 0.01) (Figure S1A) while significantly increasing the abundance of Verrucomicrobiota (*p* < 0.05) (Figure S2B). The ratio of Firmicutes to Bacteroidota (F/B) is an important indicator of gut microbiota homeostasis [30]. The DSS group had the lowest F/B ratio, while the F/B ratio in the SASP group was significantly higher than that in the DSS group (*p* < 0.05). The F/B ratio also increased in the other DSS-induced groups. Still, there were no significant differences compared to the DSS group (*p* > 0.05) (Figure 11E). Proteobacteria is associated with the development of UC, and its abundance was significantly elevated in the DSS group (*p* < 0.05) (Figure S1C). All treatment groups suppressed the increase in Proteobacteria, with the HIC and Arg groups showing the most significant effects (*p* < 0.05).

At the genus level, the top 10 most abundant genera were presented. Each treatment group had different regulatory effects. For example, *Akkermansia* shows abnormal changes in the SASP group, with a significant increase in its relative abundance (Figure 11B). Lefse analysis was used to identify genera with significant differences between groups, highlighting changes in gut microbiota and the regulatory effects of different interventions. Among the differing genera in the top 10, five key genera were identified: *Akkermansia*, *Escherichia-Shigella*, *Alistipes*, *Parabacteroides*, and *Prevotellaceae* UCG-001 (Figure 11C,D). Specifically, the abundance of *Akkermansia* significantly increases in the SASP group (Figure 11F), while the abundances of *Escherichia-Shigella* and *Alistipes* significantly increase in the DSS group (Figure 11G,H). The abundance of *Parabacteroides* significantly increases in the CFS group (Figure 11I), and the abundance of *Prevotellaceae* UCG-001 significantly increases in the HIC group (Figure 11J) (*p* < 0.05). These results indicate that HIC, CFS, and SASP treatment significantly affect the gut microbiota composition in DSS-induced colitis mice. It is important to emphasize that HIC and CFS treatment does not significantly affect the F/B, while SASP exhibits significant regulatory effects at both the phylum and genus levels, which may explain the notable influence of SASP on the overall structure of the gut microbiota. Among all treatment groups, only the Arg group neither significantly affected the F/B nor showed significant differences in genera. The relative abundances of *Akkermansia*, *Escherichia-Shigella*, *Alistipes*, *Parabacteroides*, and *Prevotellaceae* UCG-001 in this group were closest to those in the CON group, indicating that the intervention of Arg positively regulates the gut microbiota in DSS-induced colitis mice, leading to a trend of recovery toward the CON group status. This also explains why, in the clustering analysis of the overall gut microbiota structure, the Arg group is closest to the CON group.

### 3.12. Functional Prediction of Gut Microbiota Based on 16S rRNA

**Figure 12:**
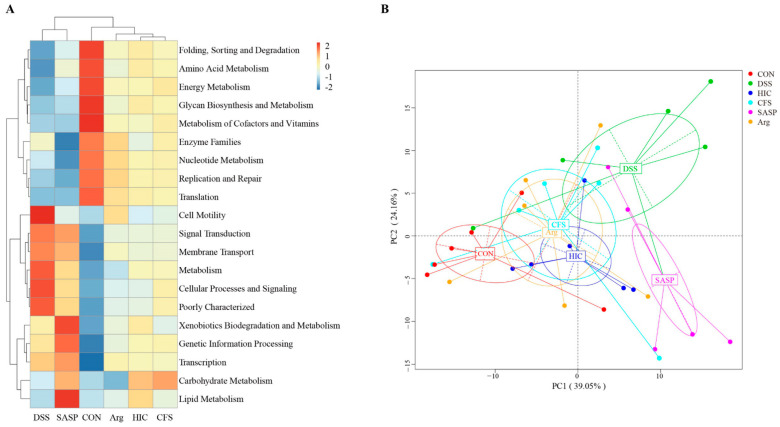
Functional prediction of gut microbiota. (**A**) Heatmap of pathway level 2. (**B**) Functional clustering PCoA analysis.

We used the PICRUSt method to predict the KEGG functional profiles of the gut microbiota based on 16S rRNA gene sequences and compiled the top 20 KEGG pathways of the mouse gut microbiota. The results indicate that the gut microbiota is involved in various important metabolic pathways, including folding, sorting and degradation, amino acid metabolism, energy metabolism, glycan biosynthesis and metabolism, and the metabolism of colactors and vitamins (Figure 12A). Pathways highly expressed in the CON group were significantly downregulated in the DSS and SASP groups. Although the expression in the Arg, HIC, and CFS groups did not recover to levels consistent with the CON group, there was also no significant downregulation. This phenomenon explains why, in the hierarchical clustering tree above the heatmap, the DSS and SASP groups are clustered together, while both of these groups are distinctly separated from the CON group. Meanwhile, the Arg, HIC, and CFS groups cluster with the CON group, with the Arg group being the most similar to the CON group. PCoA analysis further supports these findings, showing that the DSS and SASP groups are distanced from the CON group, while the Arg, HIC, and CFS groups are moving closer to the CON group (Figure 12B).

## 4. Discussion

UC is a chronic IBD with an unclear etiology, presenting symptoms such as diarrhea, rectal bleeding, and abdominal pain [31,32]. Studies have shown that *S. boulardii* and its postbiotics have a positive effect on alleviating intestinal inflammation [11,12,17]. However, studies on the therapeutic effects of different components of *S. boulardii* fermentation products on UC are limited. This study separated the fermentation products into HIC and CFS to prepare postbiotics. Arg, a key metabolite of *S. boulardii*, was also selected for its known anti-inflammatory potential [33]. Therefore, delving into Arg’s role in treating UC helps unveil the bioactivity of postbiotics. Our results demonstrate that both HIC and CFS exhibit antioxidant properties, significantly enhance the viability of Caco-2 cells, and mitigate LPS-induced inflammation and TJPs loss. Moreover, in a DSS-induced mouse colitis model, oral administration of these prebiotics restored colonic tissue damage, reduced inflammatory markers, preserved intestinal TJPs, and modulated the gut microbiota, highlighting their protective effects on gut health.

Dysfunction of the intestinal barrier is an early signal for the occurrence or development of intestinal inflammation [34]. The integrity of the intestinal barrier primarily relies on the epithelial cell membrane [35]. When intestinal epithelial cells are damaged, the integrity of the intestinal barrier is significantly weakened. TEER and ALP activity are important indicators for assessing intestinal barrier integrity [36]. ALP is a biomarker produced by the brush border cells of the small intestine [37]. The activity of ALP on both sides of the Caco-2 cell monolayer can be used to evaluate the differentiation and functional state of the cells, reflecting the integrity of the intestinal barrier [38,39]. In this study, LPS-induced damage led to a decrease in TEER and ALP activity, which was significantly reversed by HIC, CFS, and Arg (Figure 3), indicating that HIC, CFS, and Arg protect intestinal barrier function by inhibiting damage to intestinal epithelial cells. Moreover, LPS-induced damage to intestinal epithelial cells triggers the release of pro-inflammatory cytokines, which can reduce the expression of TJPs, thereby exacerbating intestinal barrier dysfunction. The results of this study show that HIC, CFS, and Arg exhibit significant inhibition of the release and gene expression of pro-inflammatory cytokines (Figure 4). Regarding subsequent TJP damage, HIC, CFS, and Arg potently counteract the downregulation of occludin and ZO-1, thereby safeguarding the integrity of intestinal tight junctions (Figure 5). This indicates that HIC, CFS, and Arg can protect intestinal barrier function through their anti-inflammatory effects.

To confirm the in vitro findings, we examined the therapeutic effects of HIC, CFS, and Arg in a mouse model of UC induced by DSS. The results showed that oral administration of HIC, CFS, and Arg significantly inhibited weight loss in UC mice, alleviated the DAI, and reduced symptoms of hematochezia and diarrhea. Moreover, oral HIC, CFS, and Arg alleviated colon shortening caused by inflammation and improved goblet cells’ atrophy and inflammatory cell infiltration in colonic tissues (Figure 6 and Figure 7), indicating their potential to improve colonic tissue damage. The spleen, as a site for immune cell proliferation and differentiation, is closely related to inflammatory responses [40,41]. Our previous studies have shown that spleens in UC mice become enlarged [17,18]. In this study, we also observed that DSS induction significantly increased the spleen index in mice, while oral administration of HIC, CFS, and Arg mitigated this change (Figure 6D), indicating their immunomodulatory effects on UC mice. Consistent with the cellular experiment results, oral HIC, CFS, and Arg reduced the expression of pro-inflammatory cytokines TNF-α, IL-1β, and IL-6 in the serum and colon of UC mice while upregulating the levels of occludin and ZO-1 in colonic tissues. It should be particularly noted that from the 8th to the 14th day of the experiment, the mice stopped drinking DSS. Although the DAI index of the DSS group began to reverse after the induction was stopped, there were still extremely significant differences compared with the mice in other groups (Figure 6C). This indicates that the intestinal tissue cannot fully recover in the short term even after the inducing factor is removed. It also highlights the necessity of exploring effective therapeutic interventions to promote intestinal repair and restore normal physiological functions.

SASP is a conventional clinical drug for the treatment of UC, composed of sulfapyridine and 5-aminosalicylic acid (5-ASA). It primarily exerts systemic anti-inflammatory and immunomodulatory effects by inhibiting the synthesis of inflammatory mediators and leukocyte infiltration [42]. In this study, we used SASP as a positive control to evaluate the effects of HIC, CFS, and Arg on UC. The results showed that SASP, HIC, CFS, and Arg exhibited similar anti-inflammatory effects (Figure 6, Figure 7, Figure 8 and Figure 9). However, in terms of gut microbiota, mice in the SASP group displayed unique characteristics. The results of this study indicated that SASP reduced microbiota diversity, indicated by a decrease in the Shannon and Simpson indices, and led to an abnormal increase in the F/B, suggesting that SASP may have an unintended regulatory effect on microbiota composition. The 5-ASA in SASP can alter the composition of the gut microbiota, with studies showing an increase in the relative abundance of Firmicutes and a decrease in the abundance of Bacteroidetes [43,44]. Combining these findings with our research results, we speculate that SASP may promote the growth of certain bacteria by altering the intestinal environment. The phylum-level composition analysis showed a significant increase in the abundance of Verrucomicrobiota in the SASP group (Figure S1), closely related to the significant increase of *Akkermansia* within this phylum (Figure 11F). *Akkermansia* is a differential genus between the SASP group and other groups and is involved in the regulation of host barrier function and immune responses. It is currently considered a promising novel probiotic for the treatment of IBD [45]. Although there is no direct evidence indicating the competitive role of *Akkermansia* in the gut, its strong colonization characteristics may suppress the survival of certain Bacteroides species. We speculate that this is a major reason for the increase in the F/B ratio following SASP administration, but the interaction mechanisms between *Akkermansia* and Bacteroidetes in the gut still need further exploration. Previous studies have shown that some UC patients cannot tolerate this drug due to adverse reactions [46,47]. Other research has indicated that while sulfasalazine has protective effects in DSS-induced colitis mice, its effectiveness is not ideal [5]. Therefore, the overall impact of SASP on the gut microbiota structure in UC patients still requires further investigation.

At the genus level, *Escherichia-Shigella* and *Alistipes* are key genera in the gut of DSS group mice, with abundances significantly higher than in other groups. Some pathogenic strains within the *Escherichia-Shigella* can trigger intestinal inflammation, leading to diarrhea and other gastrointestinal symptoms [48]. Recent studies have also identified pathogenicity in *Alistipes* bacteria related to colorectal cancer [49]. The increased abundance of Escherichia-Shigella and *Alistipes* not only reflects dysbiosis of the gut microbiota but may also be an important factor contributing to exacerbated intestinal inflammation. Therefore, in-depth research into the mechanisms of these two genera in UC will provide valuable insights into the pathogenesis of the disease and potential new therapeutic targets. In contrast, oral administration of CFS increased the abundance of beneficial gut microbiota, such as *Parabacteroides*, which are known to promote gut health by restoring barrier function and preventing metabolic diseases [50,51]. Furthermore, oral HIC increased the abundance of the beneficial genus *Prevotellaceae* UCG-001 in the intestines of colitis mice, which has shown positive effects in other studies on colitis treatment [52,53]. These findings suggest that HIC and CFS may provide therapeutic benefits by improving the composition of the gut microbiota, promoting beneficial bacteria, and maintaining intestinal barrier function.

Finally, PICRUSt analysis indicated that while oral SASP caused significant shifts in microbial function, HIC, CFS, and Arg were able to restore gut microbiota function closer to that of healthy mice. This phenomenon further confirms the unique regulatory effect of SASP on the gut microbiota, indicating that the regulatory mechanisms of HIC, CFS, and Arg differ from those of SASP. It appears that compared to SASP, HIC, CFS, and Arg may focus more on inhibiting inflammatory responses by improving the composition of the gut microbiota, they tend to make positive adjustments toward the microbiota composition observed in healthy mice. These findings provide important references for the future development of new gut microbiota modulators, particularly with potential clinical applications in treating gut-related diseases and maintaining gut health.

## 5. Conclusions

In conclusion, our study highlights the potential of *S. boulardii* fermentation products—particularly HIC, CFS, and Arg—as promising therapeutic agents for UC. By protecting the intestinal barrier function and modulating the gut microbiota, these postbiotics offer a more targeted approach to treating UC, with better preservation of the microbiota compared to traditional therapies like SASP. These findings provide important insights for the development of new gut microbiota modulators for treating intestinal diseases and lay the theoretical foundation for utilizing *S. boulardii* postbiotics as functional food supplements to enhance gut health.

## Figures and Tables

**Table 1:** Information on L-amino acids in the differential metabolites between YPD and CFS.

	Substance Name	Molecular Weight	RT [min]	Relative Quantitative
1	L-Arginine	174	8.112	543.15
2	L-Lysine	146	1.286	371.75
3	L-Tyrosine	181	2.155	356.99
4	L-Methionine	149	1.943	209.13
5	L-Phenylalanine	165	4.803	142.42
6	L-Tryptophan	204	0.577	10.23
7	L-Asparagine	132	1.34	2.66

## Data Availability

All of the data is contained within the article and the Supplementary Materials.

## Supplementary Materials

The following supporting information can be downloaded at https://www.mdpi.com/article/10.3390/antiox14060737/s1: Table S1: Disease activity index scoring criteria; Table S2: Primer sequences for human real-time fluorescent quantitative PCR; Table S3: Primer sequences for mice real-time fluorescent quantitative PCR; Table S4: The 12 differential metabolites upregulated in CFS; Figure S1: Differences in the relative abundance of Bacteroidota.

## Abbreviations

The following abbreviations are used in this manuscript:

HIC	heat-inactivated cells
CFS	cell-free supernatant
Arg	L-arginine
SASP	sulfasalazine
DPPH	2,2-diphenyl-1-picrylhydrazyl
ABTS	2,2′-azino-bis (3-ethylbenzothiazoline-6-sulfonic acid)
LPS	lipopolysaccharide
DSS	dextran sulphate sodium
TNF-α	tumor necrosis factor alpha
IL-1β	interleukin 1 beta
IL-6	interleukin 6
ZO-1	zonula occludens 1
TJPs	tight junction proteins
