# Supplementary material for: Inactivated Cells and Metabolites of Saccharomyces boulardii Alleviate Inflammation Damage in Caco-2 Monolayer Cells and Mice with Ulcerative Colitis
Source: Antioxidants (Basel). 2025 Jun 16;14(6):737. doi: 10.3390/antiox14060737 (PMC12189787; doi:10.3390/antiox14060737)
Supplement: Supplementary file 1 [file antioxidants-14-00737-s001.zip › antioxidants-3557168-supplementary.pdf]

Supplementary

Table S1. Disease activity index scoring criteria.

Score	Body weight loss (%)	Fecal traits	Hematochezia
0	≤0	Normal stools	Normal
1	1-5	Soft stools	Small amounts of blood-streaked feces
2	6-10	Loose stools	A certain amount of bloody feces
3	11-15	Semi-loose stools	Conspicuous blood-wrapped feces
4	＞15	Watery stools	Visible rectal bleeding

The DAI calculating formulais as follows: DAI = 1/3 (Body weight loss score + Fecal traits score + Hematochezia score).

Table S2. Primer sequences for human real-time fluorescent quantitative PCR.

Accession Number	Target Gene	Nucleotide Sequence of Primer (5′ to 3′) Forward	Nucleotide Sequence of Primer (5′ to 3′) Reverse
AC166162	GAPDH	AATGGGCAGCCGTTAGGAAA	GCGCCCAATACGACCAAATC
AL808143	IL-1β	AACCTCTTCGAGGCACAAGG	AGATTCGTAGCTGGATGCCG
AC112933	IL-6	CCTTCGGTCCAGTTGCCTTCT	TCTGAGGTGCCCATGCTACA
AB039224	TNF-α	GACAAGCCTGTAGCCCATGT	GGAGGTTGACCTTGGTCTGG
AC158536	occludin	TCTCCCTCCCTGCTTCCTC	GGCCAACATGAAGCCCTTTG
AC122222	ZO-1	GAAATACCTGACGGTGCTGC	GCCATCTCTTGCTGCCAAAC

Table S3. Primer sequences for mice real-time fluorescent quantitative PCR.

Accession Number	Target Gene	Nucleotide Sequence of Primer (5′ to 3′) Forward	Nucleotide Sequence of Primer (5′ to 3′) Reverse
AC006064	GAPDH	ATGGTGAAGGTCGGTGTGAA	TTTGCCGTGAGTGGAGTCAT
AC079753	IL-1β	GTCGCTCAGGGTCACAAGAA	CCACACGTTGACAGCTAGGT
AC073072	IL-6	GGAGCCCACCAAGAACGATA	GTCACCAGCATCAGTCCCAA
AB088112	TNF-α	CCCTCACACTCACAAACCAC	ACAAGGTACAACCCATCGGC
AC142525	occludin	TTTCCTGCGGTGACTTCTCC	AAAACAGTGGTGGGGAACGT
AC022613	ZO-1	GAGCAGGCTTTGGAGGAGAC	TGGGACAAAAGTCCGGGAAG

Table S4. The 12 differential metabolites upregulated in CFS.

	Substance name	log_2FC	P
1	Adenosine	1.2659445	0.0003619515
2	2-Hydroxycinnamic acid	2.169832192	0.00585564
3	Leucyl-Proline	1.421645551	0.008345128
4	N-(6-methoxypyridin-3-yl)thiophene-2-carboxamide	1.164474489	0.012043506
5	4-Hydroxybenzoic acid	2.585446494	0.0012188106
6	Thromboxane B2	1.002731495	0.016007781
7	Isobutyryl carnitine	1.007953929	0.019795064
8	Cytidine	2.800275336	0.024653871
9	9-Oxo-ODE	1.662232236	0.02681764
10	Arginine	1.554355178	0.031019883
11	1-(34-dimethylphenyl)-3-piperidinopyrrolidine-25-dione	1.189833611	0.040561459
12	Noroxycodone	2.587718305	0.041667935

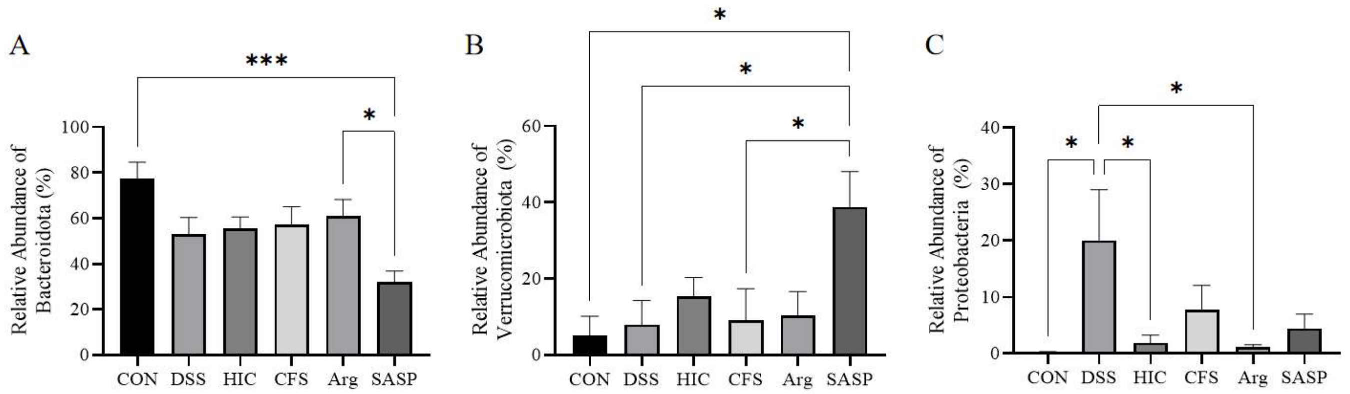

Figure S1. Differences in the relative abundance of Bacteroidota (A), Verrucomicrobiota (B), and Proteobacteria (C) at the phylum level between groups. Note: Comparison of significant differences between groups is indicated, where * $p < 0.05$, *** $p < 0.001$.
